# Effects of α- and β-Chitin on the Mechanical Properties and Molecular Interactions of Electrospun PLLA Nanofibers

**DOI:** 10.3390/polym18172127

**Published:** 2026-08-31

**Authors:** Seunghwan Choy

**Affiliations:** Digital Health Research Division, Korea Institute of Oriental Medicine, Daejeon 34054, Republic of Korea; schoy@kiom.re.kr

**Keywords:** Poly(L-lactic acid), chitin, electrospinning, mechanical property

## Abstract

Poly(L-lactic acid) (PLLA) nanofibers are promising materials for biomedical and packaging applications; however, their inherent brittleness limits applications requiring both stiffness and ductility. Here, α- and β-chitin, differing in molecular packing and hydrogen-bonding characteristics, were incorporated into electrospun PLLA nanofibers at a PLLA/chitin mass ratio of 10:1. Both chitin types enhanced the mechanical properties of PLLA, increasing tensile strength by 1.4- and 1.6-fold and Young’s modulus to 83.7 and 79.7 MPa for PLLA/α-chitin and PLLA/β-chitin, respectively. PLLA/α-chitin exhibited higher yield strength and more uniform fiber morphology, whereas PLLA/β-chitin showed substantially greater ductility and toughness with pronounced necking behavior. Thermal, spectroscopic, and diffraction analyses revealed distinct structural responses associated with the two chitin forms. α-Chitin produced a constrained hydrogen-bonded environment that restricted PLLA chain mobility. In contrast, β-chitin preserved greater chain mobility and promoted a more heterogeneous local molecular environment. Despite its lower overall crystallinity, PLLA/β-chitin exhibited a distinct secondary melting feature, suggesting localized chain organization and a limited nucleation effect rather than enhanced bulk crystallization. These findings demonstrate that chitin structure provides a practical design parameter for balancing stiffness and toughness in PLLA nanofiber composites, broadening their potential applications in the biomedical and packaging fields.

## 1. Introduction

Poly(L-lactic acid) (PLLA) has attracted considerable attention as a biodegradable and biocompatible polymer for biomedical, environmental, and sustainable materials applications [1,2]. In particular, electrospun PLLA nanofibers have been extensively investigated for use in tissue-engineering scaffolds, wound dressings, and flexible biomaterials because their fibrous architecture mimics the native extracellular matrix and provides a high surface-area-to-volume ratio [3,4]. Despite these advantages, the inherent brittleness and limited toughness of PLLA remain major obstacles to its broader practical use, particularly in applications requiring sustained mechanical integrity and deformation resistance [5,6]. To address these limitations, various modification and reinforcement strategies, including polymer blending, plasticization, copolymerization, incorporation of reinforcing fillers, and other toughening approaches, have been explored to improve the mechanical performance of PLA/PLLA-based materials [7,8,9,10,11,12]. More specifically, recent studies have reported various approaches to reinforce or toughen electrospun PLLA-based materials, including self-reinforcement, the incorporation of halloysite nanotubes, and polymer blending strategies [13,14,15,16].

Among naturally derived reinforcing materials, chitin has emerged as a promising candidate due to its high crystallinity, biodegradability, biocompatibility, and abundant hydroxyl and acetamide functional groups capable of forming intermolecular interactions with polymer matrices [17,18]. Chitin exists in two principal crystalline forms that differ fundamentally in molecular packing: α-chitin, found in crustacean shells and insect cuticles, adopts an antiparallel chain arrangement with a high density of intermolecular hydrogen bonds, resulting in a dense and rigid crystal structure; β-chitin, found primarily in squid pens, adopts a parallel chain arrangement with a lower hydrogen-bond density, yielding a more open structure with greater free volume and solvent accessibility. These structural differences are expected to strongly influence not only the dissolution and processing behavior of chitin but also its interaction with a surrounding polymer matrix and its consequent reinforcing mechanism [19,20,21].

Previous studies on PLLA/chitin composites have primarily focused on the general reinforcing effect of chitin without distinguishing between α- and β-chitin, treating chitin mainly as a reinforcing filler or based on its functional groups [22,23]. Furthermore, several studies have demonstrated that the distinct crystal structures of α- and β-chitin can result in different interactions with polymer matrices [24,25]. In electrospun collagen/chitin composites, β-chitin exhibited more favorable intermolecular interactions with collagen and greater tensile strength than α-chitin, demonstrating that the molecular arrangement of chitin can significantly influence composite structure and mechanical properties [25]. Therefore, understanding how different types of chitins affect the structural and mechanical behavior of electrospun PLLA composites is essential for designing high-performance nanofibrous materials.

In this study, high-purity α- and β-chitin were incorporated into PLLA nanofibers by electrospinning using an optimized hexafluoroisopropanol (HFIP)/trifluoroacetic acid (TFA) solvent system. The resulting composites were systematically characterized to elucidate the relationships between their mechanical properties, molecular interactions, and spectroscopic characteristics. We hypothesized that the distinct molecular packing structures of α- and β-chitin would increase the Young’s modulus of the composites, whereas differences in chitin–PLLA interactions and their effects on PLLA chain organization would result in contrasting post-yield deformation behaviors. In particular, β-chitin was expected to impart greater ductility and toughness than α-chitin by facilitating the formation of a more structurally adaptive composite network. The findings provide a structural basis for selecting the type of chitin to tailor the balance between stiffness and toughness in PLLA-based nanofiber composites.

## 2. Materials and Methods

### 2.1. Materials

PLLA (Cat. No. 18402, Polysciences, Inc., Warrington, PA, USA; M_w_ ≈ 80,000–100,000) was used as the base polymer, while α- and β-chitin were incorporated as reinforcing additives to enhance the mechanical properties of the electrospun nanofibers. High-purity α-chitin (C9752) was purchased from Sigma-Aldrich (St. Louis, MO, USA), and β-chitin (ARA-170124FC) was obtained from AraBio (Seoul, Republic of Korea). Hexafluoroisopropanol (HFIP, A1247, Alfa Aesar) and trifluoroacetic acid (TFA, L06374, Alfa Aesar) were used as a binary solvent system for polymer dissolution.

### 2.2. Preparation of PLLA/Chitin Solutions

To optimize the solvent conditions for chitin dissolution and subsequent electrospinning, various HFIP/TFA ratios were investigated. After identifying the solvent composition range that allowed complete dissolution of chitin, the HFIP/TFA ratio was further optimized based on the spinnability of the resulting solutions. An HFIP/TFA ratio of 85:15 (*v*/*v*) was selected because it provided stable electrospinning and reproducibly produced uniform, bead-free nanofibers. The total polymer concentration was fixed at 11% (*w*/*v*). For the PLLA/chitin composite, a mass ratio of 10:1 (PLLA–chitin) was utilized.

### 2.3. Electrospinning

The polymer solution was loaded into a syringe equipped with a 26-gauge stainless-steel needle. The distance between the needle tip and the collector was maintained at approximately 8 cm. A voltage of 10–20 kV was applied to generate the polymer jet, and the solution feed rate was controlled at 0.25 mL/h. All electrospinning experiments were conducted at 23.5 °C under controlled relative humidity conditions of 40 ± 2%.

### 2.4. Scanning Electron Microscopy (SEM)

The morphologies of the electrospun nanofibers and solvent-cast films were characterized using scanning electron microscopy (SEM, JSM-6010LV, JEOL, Tokyo, Japan) after gold sputter coating. The average fiber diameter was determined by analyzing 100 randomly selected fibers using ImageJ software (version 1.54f, National Institutes of Health, Bethesda, MD, USA).

### 2.5. Fourier-Transform Infrared Spectroscopy (FT-IR)

Fourier-transform infrared spectroscopy (FT-IR, Nicolet™ iS™ 50 FT-IR Spectrometer, Thermo Fisher Scientific Co., Ltd., Waltham, MA, USA) was performed at a range of 4000 to 400 cm^−1^ to investigate the molecular interactions between PLLA and chitin in the electrospun nanofibers. The formation of intermolecular interactions was evaluated by collecting the characteristic absorption bands.

### 2.6. Differential Scanning Calorimetry (DSC)

Differential scanning calorimetry (DSC, DSC 4000, PerkinElmer, Waltham, MA, USA) was employed to investigate the thermal behavior of the electrospun PLLA/chitin nanofibers. Thermal transitions and changes in crystalline structure resulting from the incorporation of α- and β-chitin were analyzed from the obtained thermograms. Samples were equilibrated at 30 °C for 15 min and then heated to 300 °C at a rate of 10 °C/min under a nitrogen atmosphere. The degree of crystallinity (*X*_c_) of PLLA was calculated according to the following equation:

*X*_c_ (%) = [(Δ*H*_m_ − Δ*H*_cc_)/(*w*_PLLA_ × Δ*H*°_m_)] × 100 (1)

where Δ*H*_m_ and Δ*H*_cc_ are the melting and cold-crystallization enthalpies (J/g), respectively; *w*_PLLA_ is the weight fraction of PLLA in the composite; and Δ*H*°_m_ is the melting enthalpy of 100% crystalline PLLA, taken as 93.0 J/g [26].

### 2.7. Uniaxial Tensile Test

Mechanical properties of the electrospun mats were evaluated using a universal testing machine (UTM, 3344, Instron, Norwood, MA, USA) at a crosshead speed of 10 mm/min. The specimens were cut into strips measuring 5 mm × 20 mm. Approximately 5 mm at each end of the specimen was secured by the grips, resulting in an initial gauge length of 10 mm. Sample thickness was measured using a digital micrometer and averaged over five positions. Tensile toughness was calculated as the area under the engineering stress–strain curve from the beginning of loading to fracture, according to $U=\int_{0}^{\varepsilon_{f}}\sigma d\varepsilon$, where U is the tensile toughness, σ is the engineering stress, and $\varepsilon_{f}$ is the strain at fracture.

### 2.8. X-Ray Diffraction (XRD) Analysis

XRD measurements were conducted using a D/MAX-2500/PC diffractometer (Rigaku Co. Ltd., Tokyo, Japan) with Ni-filtered Cu Kα radiation at an operating voltage of 40 kV and a current of 100 mA. The diffraction data were recorded over a 2θ range of 5° to 50° at a scan rate of 1°/min.

### 2.9. Preparation of Schematic Figure

**Figure 1:**
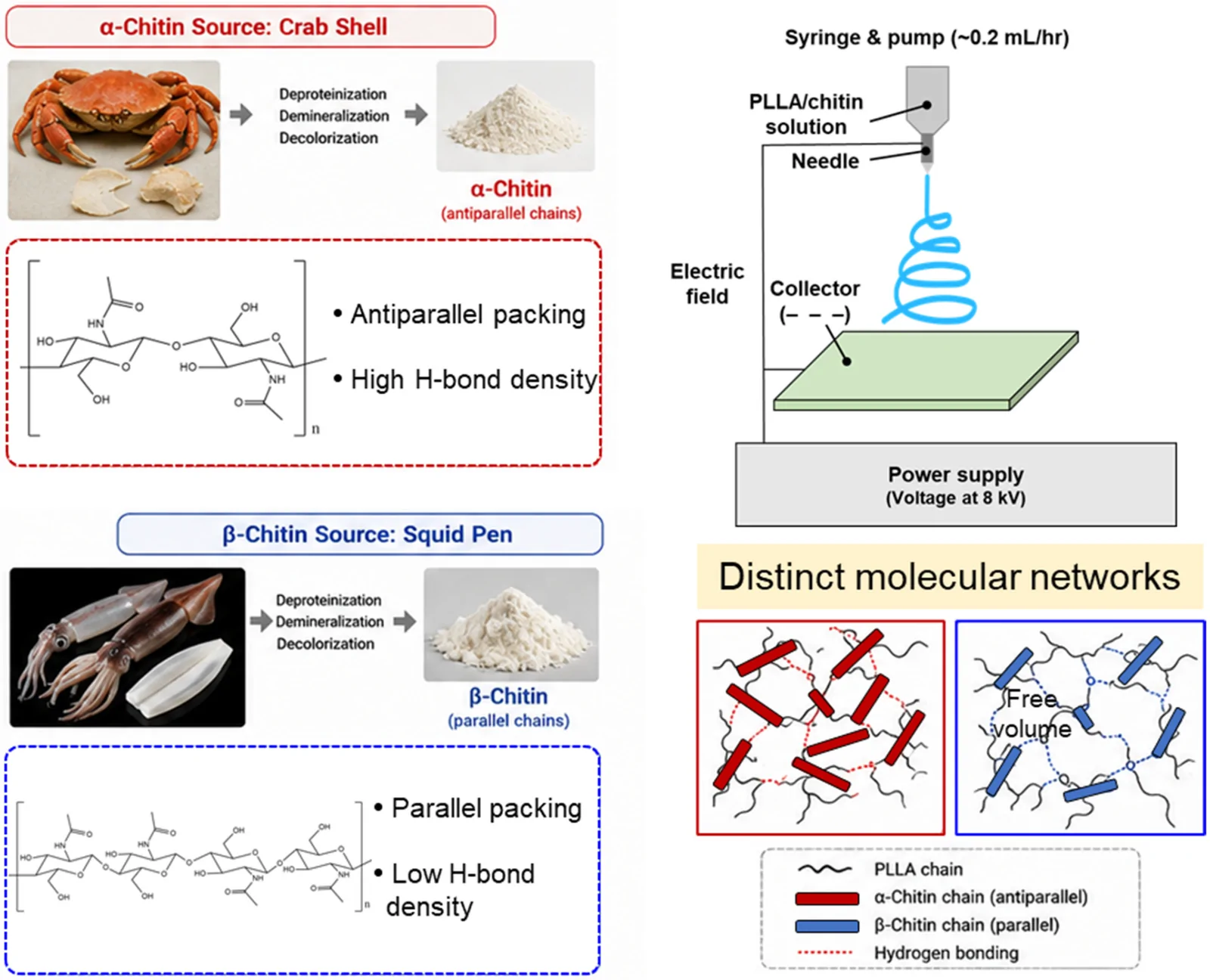
Schematic figures of electrospinning of PLLA/chitin blending and its distinct molecular networks.

Figure 1 was prepared and assembled by the author. The crab and squid illustrations representing the sources of α-chitin and β-chitin, respectively, were generated with the assistance of OpenAI ChatGPT (GPT-5.6 Sol) using its image-generation functionality. All other components of Figure 1, including the molecular structures, electrospinning schematic, labels, and schematic representations of molecular networks, were prepared and assembled by the author. Generative AI was used solely for the crab and squid illustrations and was not used to generate, modify, or analyze any experimental data or results presented in this study.

## 3. Results and Discussion

### 3.1. Molecular Architecture and Dissolution Behavior of α- and β-Chitin

The electrospinning process of PLLA/chitin composite nanofibers, along with the distinct molecular architectures of α-chitin and β-chitin, is schematically illustrated in Figure 1. α-chitin is characterized by an antiparallel packing arrangement and a high density of hydrogen bonds, which inherently results in a dense crystalline structure. Conversely, β-chitin adopts a parallel packing configuration with a lower hydrogen-bond density, creating a relatively larger free volume [27]. These structural discrepancies dictate both the interaction potential with the PLLA matrix and the susceptibility to the solvent system. Specifically, the expanded molecular arrangement of β-chitin facilitates the penetration of PLLA chains, thereby promoting the formation of robust intermolecular hydrogen bonds. On this basis, we hypothesized that the crystal structure of chitin would enhance the Young’s modulus of the composites, while the interaction between PLLA and chitin would improve their ductility, together conferring superior toughness on β-chitin-based composites relative to their α-chitin counterparts.

**Figure 2:**
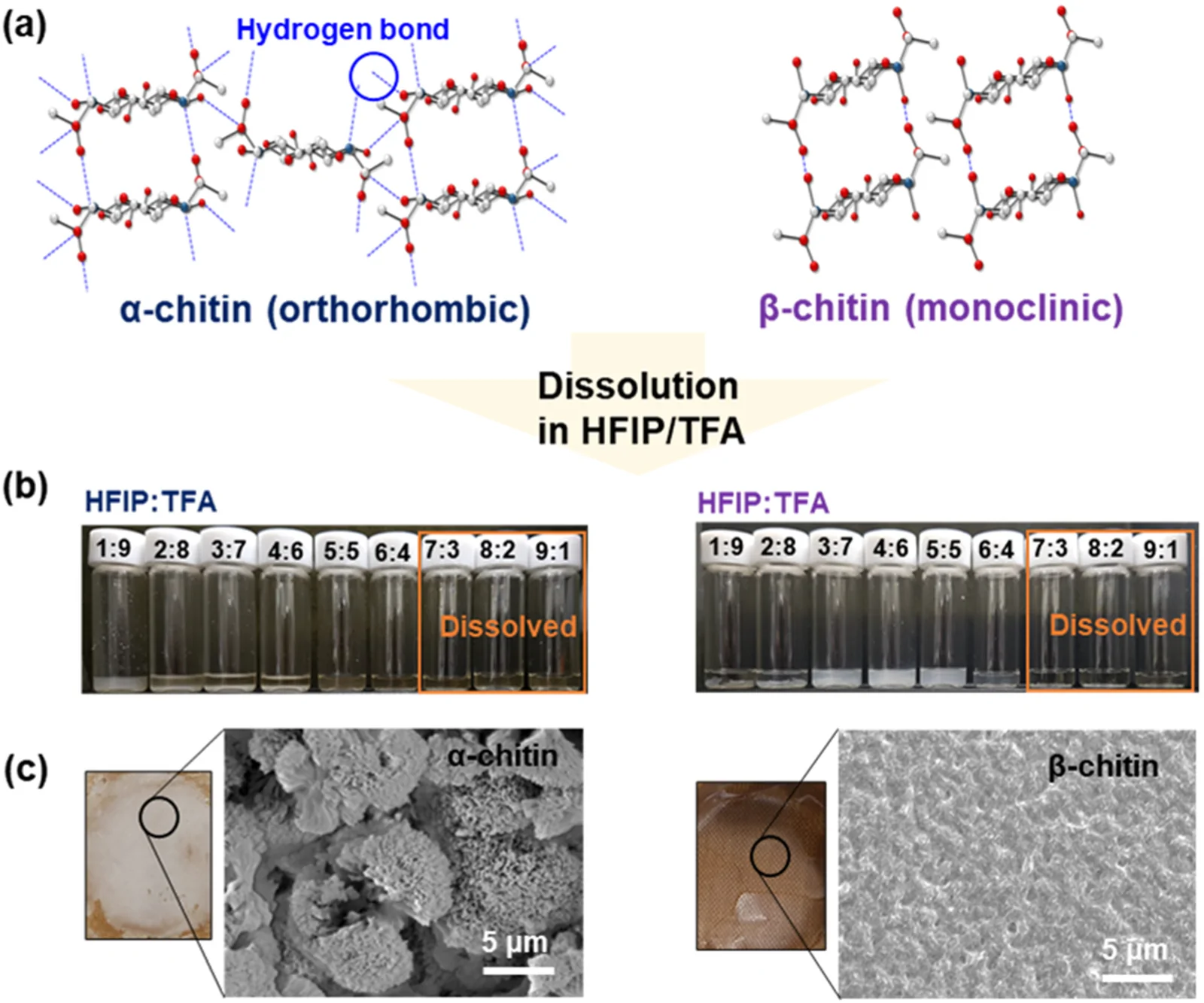
(**a**) Two types of chitin structures and (**b**) solubility test in HFIP/TFA binary solvent system. (**c**) Corresponding SEM micrographs of neat α- and β-chitin at 85:15 (HFIP:TFA) depending on their different molecular packing structures and solvent susceptibilities.

To examine how the type of chitin influences their interactions with PLLA, α- and β-chitin were employed as reinforcing components in PLLA-based composites. As the two chitin forms possess distinct molecular packing arrangements and hydrogen-bonding networks, they exhibited markedly different dissolution behaviors and miscibility characteristics in solvent systems (Figure 2a). Prior to composite fabrication, the solvent composition suitable for chitin dissolution was optimized by evaluating α-chitin solubility in mixtures of HFIP/TFA (Figure 2b). The results indicated that the addition of a limited amount of acidic solvent to a fluorinated alcohol significantly improved chitin solubilization. Complete dissolution of chitin was observed at HFIP/TFA ratios of 7:3, 8:2, and 9:1 (*v*/*v*). However, complete dissolution alone did not necessarily ensure optimal electrospinning performance. Therefore, after identifying the solvent composition range that enabled complete dissolution, the HFIP/TFA ratio was further optimized by evaluating the spinnability of the resulting solutions. Among the tested conditions, an HFIP/TFA ratio of 85:15 (*v*/*v*) provided stable jet formation and consistently produced uniform, bead-free nanofibers. Accordingly, this solvent composition was selected for all subsequent electrospinning experiments. After solvent casting under identical conditions on Teflon substrates, neat α- and β-chitin solutions generated films with clearly different surface characteristics (Figure 2c). SEM analysis revealed that α-chitin produced an opaque film containing densely aggregated granular domains with an average feature size of approximately 5 μm. In contrast, β-chitin formed a transparent film with a comparatively homogeneous and smooth surface texture. These observations suggest that the differences in molecular orientation and crystalline organization between the two types of chitins strongly affect their dissolution behavior and structural reconstruction during solvent evaporation.

Structurally, α-chitin adopts an orthorhombic crystalline organization based on antiparallel chain packing, whereas β-chitin exhibits a monoclinic organization based on parallel chain packing [28,29,30]. By comparison, β-chitin adopts a monoclinic system with a single-chain unit cell arising from parallel chain packing, with a b-axis (chain-repeat) spacing of 9.26 Å. Although this value is numerically smaller than the b-axis of α-chitin, it reflects the periodicity of a single-chain repeat rather than a more open or less densely packed structure and should not itself be taken as evidence of greater structural accessibility. The critical structural distinction instead lies in the hydrogen-bonding network connecting adjacent chitin sheets: the antiparallel packing of α-chitin supports an extensive network of both intra-sheet and inter-sheet hydrogen bonds, whereas the parallel arrangement of β-chitin precludes inter-sheet hydrogen bonding, leaving neighboring sheets held together only by comparatively weak van der Waals and dipole interactions. The absence of inter-sheet hydrogen bonds rather than any difference in axial spacing renders the β-chitin lattice far less constrained in the direction of sheet stacking. This allows for greater interlamellar accessibility, easier solvent penetration, and a greater capacity for chain rearrangement compared to the tightly interlocked, antiparallel structure of α-chitin. This looser inter-sheet architecture is expected to confer enhanced deformability and interfacial mobility once β-chitin is incorporated into the PLLA matrix. Based on these structural distinctions, we anticipated that incorporating α- or β-chitin into PLLA would lead to different fiber-assembly behaviors and, ultimately, different macroscopic mechanical properties in the resulting composites.

### 3.2. Fiber Morphology of Electrospun Composites

**Figure 3:**
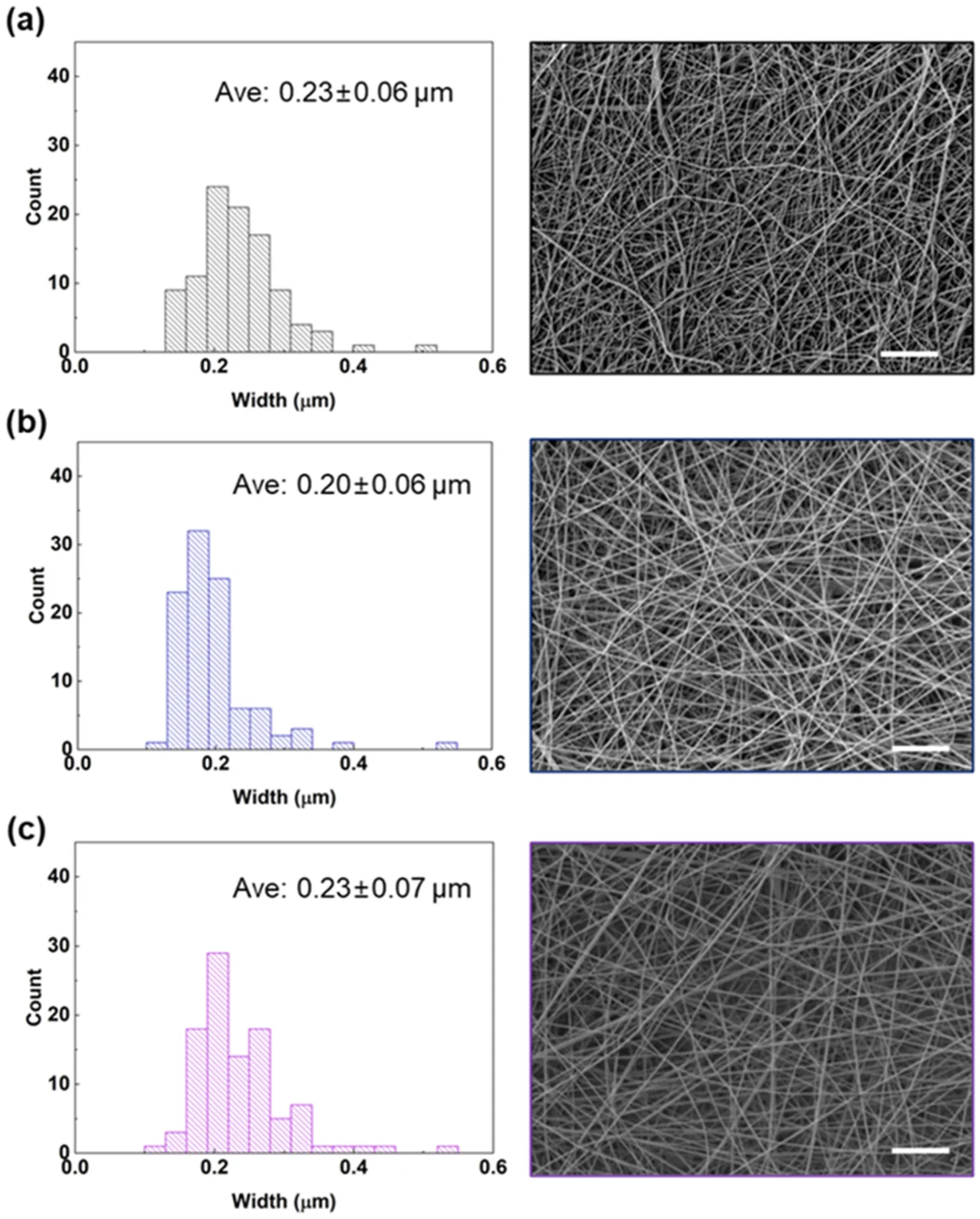
Average fiber diameter distributions of electrospun (**a**) PLLA, (**b**) PLLA/α-chitin, and (**c**) PLLA/β-chitin, along with their corresponding SEM images (*n* = 100). Scale bars = 5 μm.

The morphology and diameter distributions of electrospun PLLA, PLLA/α-chitin, and PLLA/β-chitin nanofibers are presented in Figure 3. All three groups formed continuous, bead-free fibers, though clear differences in morphology and diameter distribution emerged depending on the type of chitin incorporated. Pristine PLLA fibers were relatively uniform, with an average diameter of approximately 0.23 μm; PLLA/β-chitin fibers showed a similar average diameter, albeit with slight variation in alignment and surface texture (Figure 3a,c). PLLA/α-chitin fibers, by contrast, exhibited a reduced average diameter of approximately 0.20 μm (Figure 3b). This reduction in average fiber diameter, together with the comparatively narrow diameter distribution observed for PLLA/α-chitin, is likely associated with the increased extensional viscosity and restricted chain mobility imparted by the densely and uniformly hydrogen-bonded α-chitin network, which promotes stronger, more consistent jet stretching during electrospinning [31]. This narrow and homogeneous diameter distribution is consistent with α-chitin forming a spatially uniform, molecularly integrated network with PLLA rather than inducing localized crystallization or phase separation. β-Chitin, with its more open and flexible crystal structure, likewise achieved effective molecular integration with PLLA, yielding an average fiber diameter comparable to that of pristine PLLA; however, PLLA/β-chitin exhibited a broader diameter distribution than PLLA/α-chitin, which may reflect a more heterogeneous local molecular environment within the fibers compared with the more uniformly bonded PLLA/α-chitin system.

### 3.3. Thermal Properties

**Figure 4:**
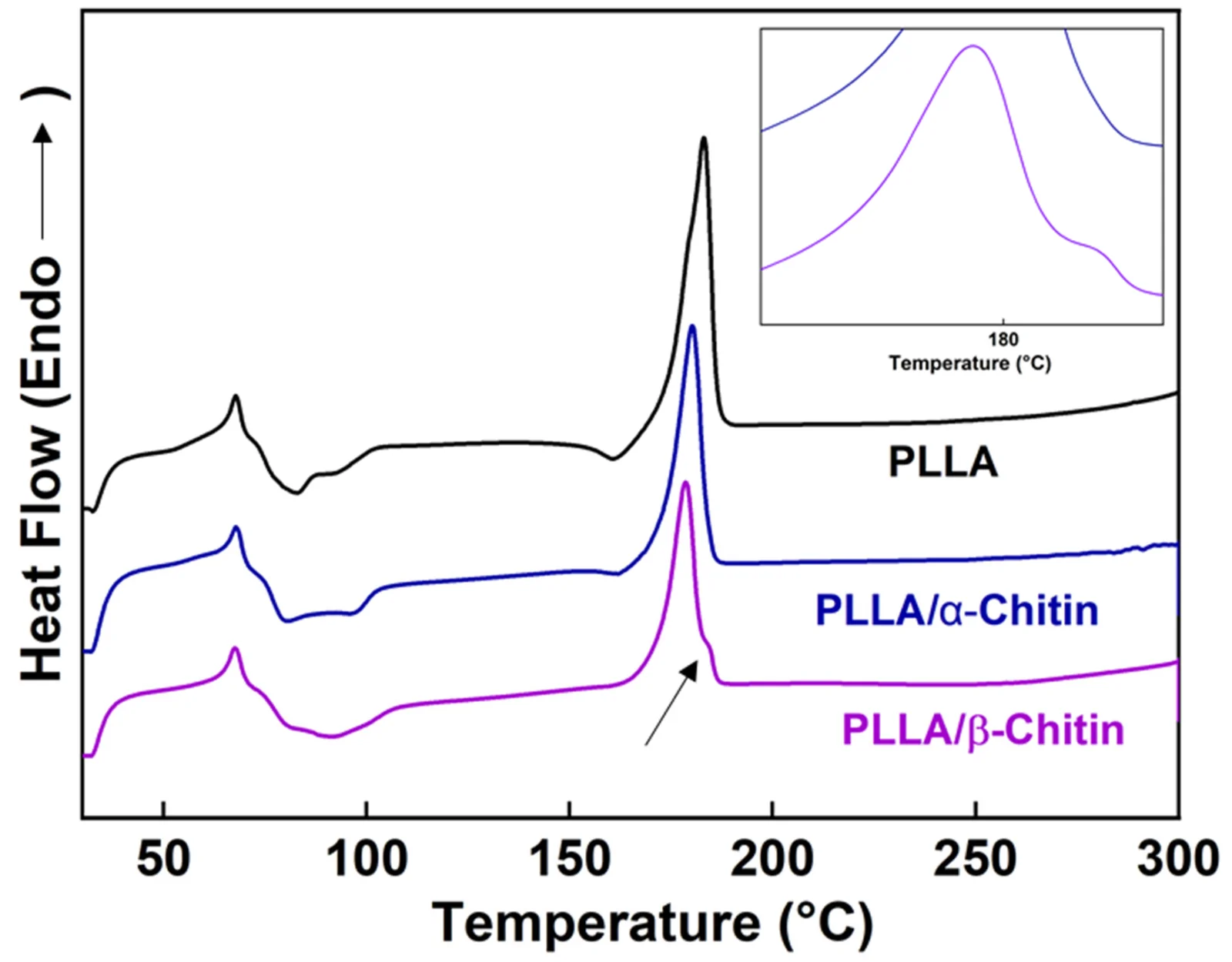
Differential scanning calorimetry thermograms of PLLA blended with α- and β-chitin (arrow: growth of new peaks).

Figure 4 shows the thermal behavior of the composites, as assessed by DSC. The resulting thermograms reveal clear differences in the crystallization and melting behavior of the different groups. All three samples exhibited a small endothermic feature near 64 °C, which is consistent with the glass transition of PLLA convolved with an enthalpic relaxation peak that is characteristic of the rapidly quenched, non-equilibrium glassy state produced during electrospinning. This was followed by a broad cold-crystallization exotherm (T_cc_) at 77–88 °C.

All three samples subsequently displayed a common main melting endotherm (T_m_) between 175 and 179 °C (Table 1). Quantifying the melting and cold-crystallization enthalpies showed that overall crystallinity decreased substantially upon chitin incorporation, in the following order: PLLA (X_c_ ≈ 49%) > PLLA/α-chitin (X_c_ ≈ 23%) > PLLA/β-chitin (X_c_ ≈ 14%). This indicates that both chitin polymorphs act as physical obstacles that interrupt the strain-induced crystallization ordinarily promoted by jet stretching during electrospinning of pure PLLA, rather than enhancing bulk crystallinity as initially hypothesized. As established in Section 3.1, α-chitin disperses poorly in the PLLA solution because its dense inter-sheet hydrogen bonding limits solvent penetration, producing coarse, locally aggregated domains rather than a finely dispersed filler. PLLA far from these α-chitin aggregates can therefore still crystallize by the same strain-induced mechanism as neat PLLA. This results in a higher overall X_c_ for PLLA/α-chitin than for PLLA/β-chitin, despite the presence of filler in both. It is notable that only PLLA/β-chitin exhibited an additional, distinct endothermic shoulder near 181 °C, superimposed on the shared main melting peak. Neither neat chitin polymorph is expected to show a thermal transition in this range. Chitin typically exhibits only a broad, low-temperature endotherm associated with moisture loss and undergoes decomposition well above 300 °C [32]. This peak is therefore not attributable to chitin itself. Instead, it is tentatively attributed to the melting of a locally distinct PLLA crystalline population within the composite. Deconvolution of this feature isolated a discrete secondary endotherm of approximately 2.7 J/g out of the total melting enthalpy of approximately 44.0 J/g for PLLA/β-chitin. The emergence of this feature only in the β-chitin composite suggests that β-chitin may provide localized sites that facilitate PLLA chain organization and the formation of a small, distinct crystalline population. Importantly, this localized effect should be distinguished from the overall crystallinity of the composite, which remained the lowest for PLLA/β-chitin (X_c_ ≈ 14%). Thus, β-chitin does not promote bulk PLLA crystallization but may exert a limited local nucleation effect within an otherwise predominantly low-crystallinity matrix. The absence of a corresponding endothermic feature in PLLA/α-chitin further suggests different local chain organizations around the two chitin structures. These thermal differences provide direct evidence for the two-phase, nucleated semicrystalline structure proposed above for PLLA/β-chitin and for the moderately crystalline, densely and uniformly hydrogen-bonded network proposed for PLLA/α-chitin. This links the mechanical differences discussed in Section 3.4 to distinct chitin-induced crystallization behavior.

### 3.4. Mechanical Properties

**Figure 5:**
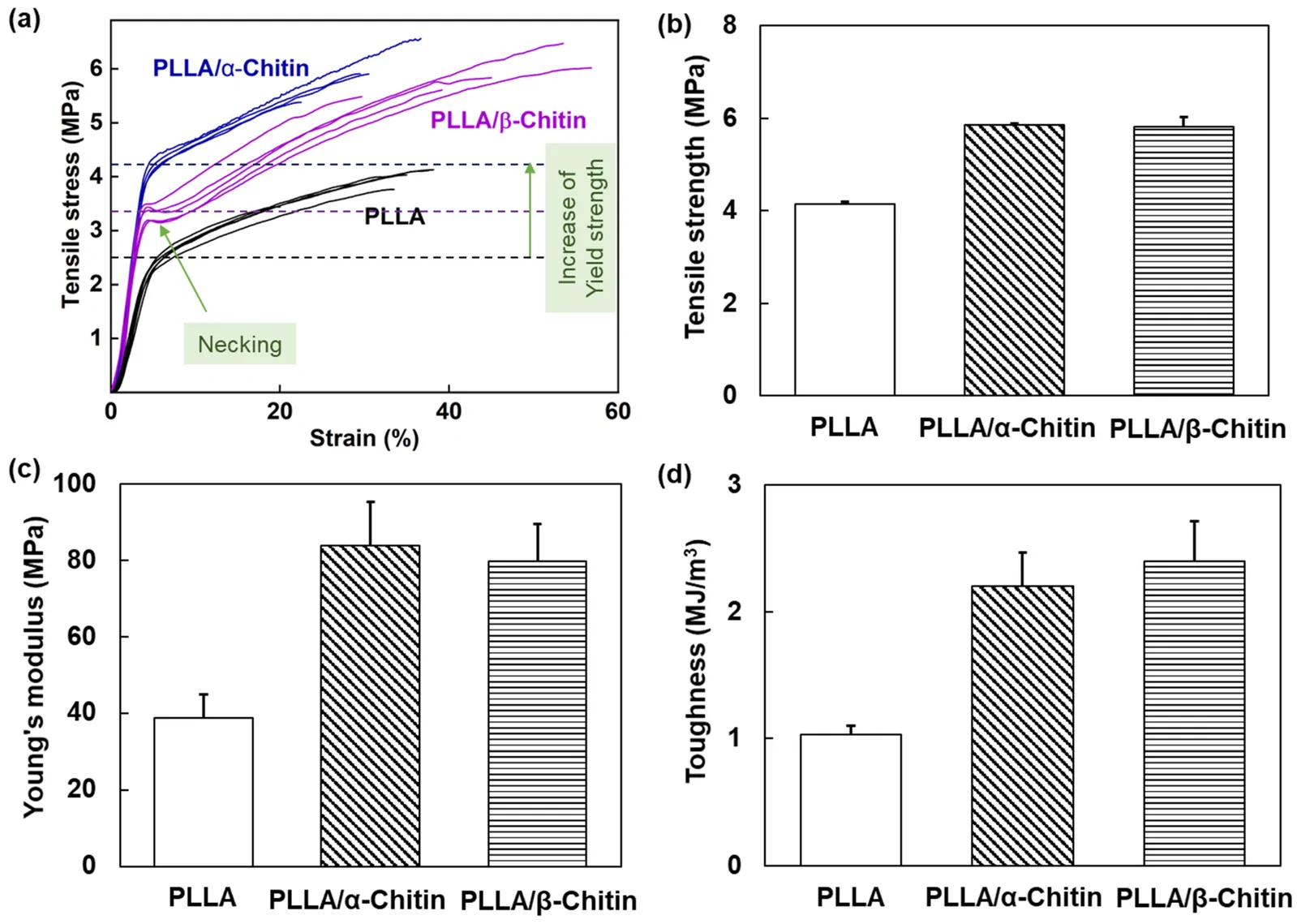
Mechanical behaviors of electrospun mats: (**a**) representative stress–strain curves of PLLA and PLLA/chitin composites; (**b**) tensile strength; (**c**) Young’s modulus; (**d**) toughness. The dashed lines indicate the yield points.

The mechanical performance of the fabricated nanofiber membranes, evaluated through tensile testing, is presented in Figure 5. The incorporation of α- and β-chitin into the PLLA matrix resulted in distinctly different mechanical responses despite both composites exhibiting enhanced stiffness compared to pristine PLLA (Figure 5a). Pure PLLA displays a characteristic low yield strength followed by rapid yielding; however, the incorporation of chitin effectively mitigates these behaviors. Specifically, the β-chitin-reinforced samples show a notable enhancement in yield strength and a delayed onset of necking.

The tensile strength of the PLLA/α-chitin and PLLA/β-chitin composites was enhanced relative to that of pristine PLLA, with the increase being approximately 1.4-fold for PLLA/α-chitin and 1.6-fold for PLLA/β-chitin (Figure 5b). In particular, the Young’s modulus increased in both PLLA/α-chitin (83.7 ± 11.5 MPa) and PLLA/β-chitin (79.7 ± 9.9 MPa) composites, indicating that both chitins effectively contributed to stress transfer within the electrospun fibrous network (Figure 5c). However, the deformation behavior after yielding differed significantly depending on the crystal structure of the incorporated chitin. PLLA/α-chitin exhibited the highest yield strength of 4.2 ± 0.04 MPa among the tested groups, whereas PLLA/β-chitin showed substantially improved ductility (strain of 47%) accompanied by pronounced necking behavior, resulting in a higher toughness of 2.4 ± 0.3 MJ/m^3^ (Figure 5d). A densely packed antiparallel molecular arrangement may strongly restrict the mobility of surrounding PLLA chains. During electrospinning, such rigid interfacial interactions likely promoted enhanced chain alignment and dense molecular packing along the fiber axis. This interpretation is supported by the reduced average fiber diameter and narrower diameter distribution observed in the PLLA/α-chitin group. The smaller and more homogeneous fiber morphology suggests that α-chitin increased the effective extensional viscosity and stabilized jet stretching during electrospinning. Consequently, the constrained amorphous chain mobility and enhanced orientation probably delayed the initiation of chain slippage under tensile loading, thereby increasing the yield strength [33]. In contrast, β-chitin, which has a less constrained structure, may provide greater free volume and interfacial flexibility within the PLLA matrix. As a result, PLLA chains surrounding β-chitin could retain a higher degree of mobility and rearrangement capability during deformation. This molecular environment likely facilitated localized plastic deformation processes such as chain sliding, disentanglement, and stress-induced molecular reorientation. The pronounced necking behavior observed in PLLA/β-chitin supports this interpretation, suggesting that the composite was capable of sustaining stable plastic deformation rather than undergoing brittle fracture immediately after yielding. Therefore, β-chitin may function not only as a reinforcing filler but also as a deformable interphase modifier that preserves ductile deformation mechanisms while still enhancing stiffness. These findings demonstrate that the structure of chitin plays a critical role in regulating the molecular packing, chain dynamics, and macroscopic mechanical behavior of electrospun PLLA composites.

### 3.5. FT-IR Analysis

**Figure 6:**
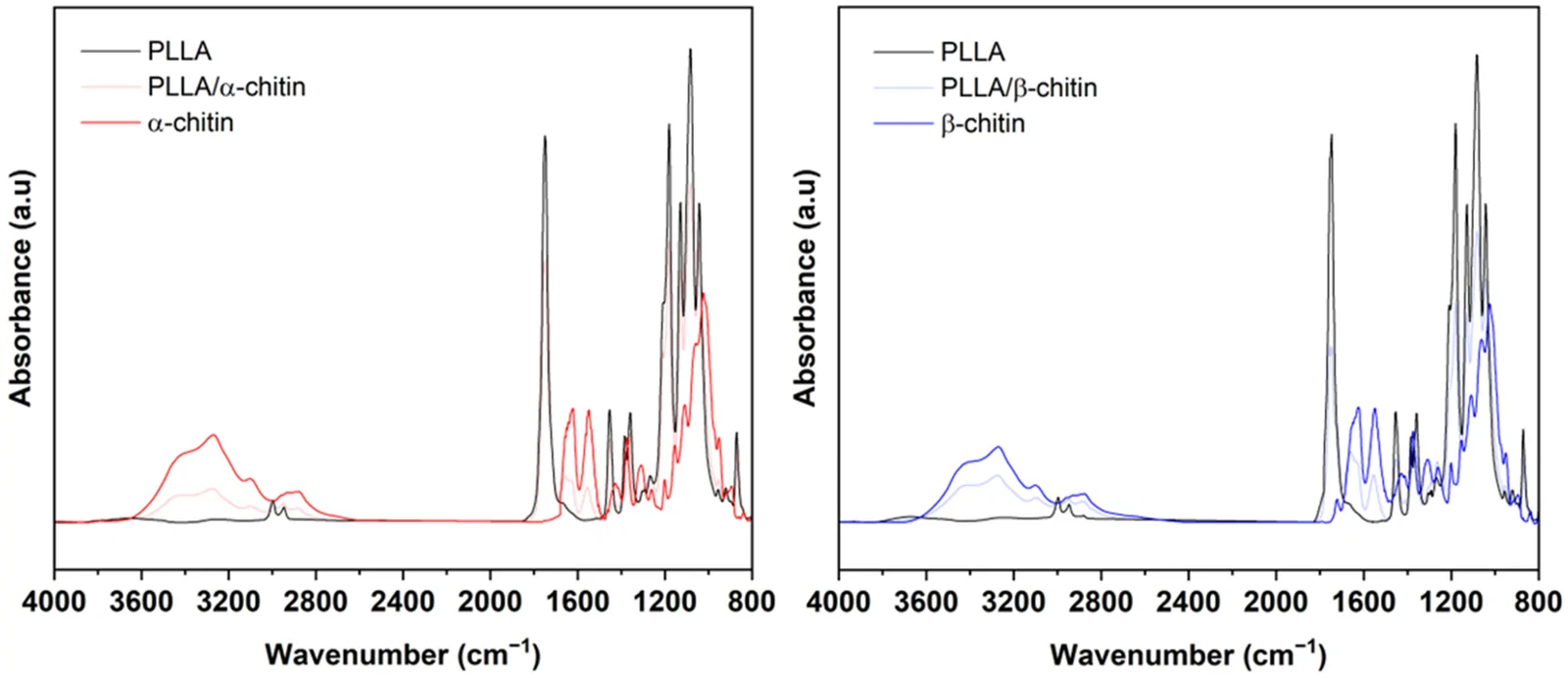
FT-IR spectra of pristine α-chitin and β-chitin together with those of PLLA, PLLA/α-chitin, and PLLA/β-chitin.

FT-IR analysis revealed distinct changes in the vibrational characteristics of PLLA upon incorporation of α- and β-chitin, reflecting differences in intermolecular interaction and crystalline structure modulation between the two types of chitin (Figure 6). The characteristic FT-IR bands and their corresponding assignments are summarized in Table 2. The characteristic C–H stretching bands of PLLA at 2998 and 2945 cm^−1^ decreased in intensity after the addition of either chitin form, indicating restricted PLLA chain mobility arising from intermolecular interaction with chitin. Similarly, the C–H bending bands at 1454 and 1362 cm^−1^ decreased in both chitin-containing systems [34], further supporting the formation of intermolecular hydrogen bonds and altered chain packing. Several spectral shifts occurred irrespective of chitin crystal form: the carbonyl (C=O) stretching band of PLLA shifted from 1749 to 1755 cm^−1^ upon addition of either α- or β-chitin [35], reflecting changes in the local chemical environment around the ester groups, while the C–O stretching band shifted from 1180 to 1185 cm^−1^ in both systems, implying altered chain conformation and crystallinity. However, other spectral changes were strongly dependent on the type of chitin. The C–OH bending vibration at 1081 cm^−1^ [35], present in both neat PLLA and the α-chitin composite, shifted to 1065 cm^−1^ only in the PLLA/β-chitin, while the C–COO stretching band at 867 cm^−1^ [36], retained in neat PLLA and the PLLA/α-chitin, disappeared or was strongly suppressed in the β-chitin composite. It can be posited that the observed shifts in β-chitin do not merely signify an increased propensity for hydrogen bonding. Rather, these shifts are more likely to be indicative of a distinct local chain conformation and packing environment surrounding the PLLA ester groups.

Therefore, both α- and β-chitin restrict PLLA chain mobility and interact with PLLA through intermolecular hydrogen bonding, as reflected in the common C–H, C=O, and C–O band changes observed for both composites. The β-chitin composite exhibited a locally distinct chain conformation and packing environment in the surrounding PLLA, consistent with the localized structural organization suggested by the DSC results. These FT-IR results therefore provide molecular-level evidence for different local chemical environments in the PLLA/α-chitin and PLLA/β-chitin composites, supporting their divergent mechanical and thermal behaviors.

### 3.6. XRD Analysis

**Figure 7:**
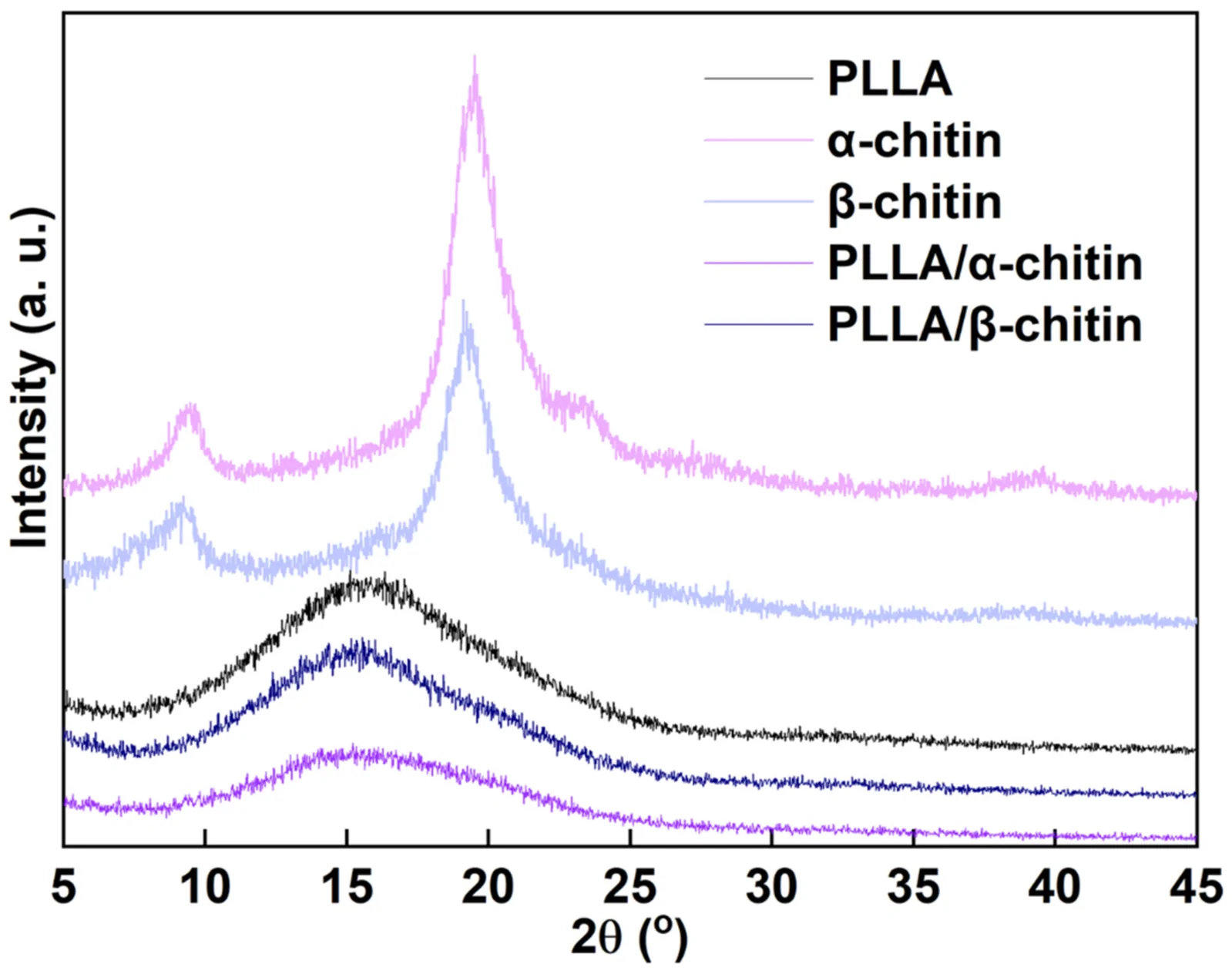
X-ray diffraction patterns of pristine α-chitin and β-chitin and electrospun PLLA, PLLA/α-chitin, and PLLA/β-chitin mats.

The crystalline structures of pristine α-chitin, pristine β-chitin, electrospun PLLA, PLLA/α-chitin, and PLLA/β-chitin were examined by XRD (Figure 7). The pristine α- and β-chitin exhibited distinct crystalline diffraction profiles, including characteristic low-angle reflections at approximately 9.5° and 8.9°, respectively, consistent with their different crystalline organizations. In contrast, the electrospun PLLA and PLLA/chitin composites were dominated by broad diffraction profiles centered at approximately 15–17°, indicating predominantly amorphous or poorly ordered structures [37]. No clearly resolved chitin-specific crystalline reflections were identified in either PLLA/α-chitin or PLLA/β-chitin. In particular, the weak feature around 20–21° was not sufficiently distinct to permit an unambiguous assignment to a specific chitin reflection. Therefore, this feature was not considered direct evidence for the retention of a specific chitin crystalline structure within the electrospun composites. The absence of clearly resolved chitin-specific reflections in both composites indicates that the long-range crystalline organization characteristic of the pristine chitins was not directly resolved after incorporation into the electrospun PLLA matrix. This may arise from disruption of long-range chitin ordering during solution processing and electrospinning, the relatively low chitin content in the composites, and/or overlap with the broad diffraction contribution of the PLLA matrix. Therefore, the XRD data alone cannot distinguish the detailed local organization of α- and β-chitin within the electrospun fibers. This interpretation is consistent with the mechanical data, in which PLLA/α-chitin exhibited the highest yield strength and a reduced, more uniform fiber diameter, both indicative of restricted amorphous chain mobility and enhanced chain orientation arising from dense interfacial hydrogen bonding, rather than from the retention of a separate, well-ordered crystalline chitin phase. Although XRD did not resolve distinct chitin-related long-range crystalline structures in the electrospun composites, this does not exclude localized or short-range molecular organization that may not produce distinct diffraction peaks. Accordingly, the XRD results were interpreted together with the DSC and FT-IR results rather than as independent evidence of a specific retained chitin crystalline phase. The combined results suggest that α- and β-chitin generate different local molecular environments within PLLA, which are associated with their distinct mechanical responses.

## 4. Conclusions

In this study, α- and β-chitin were systematically compared as reinforcing fillers in electrospun PLLA nanofiber composites to elucidate how chitin structure governs fiber morphology and mechanical performance. The different molecular arrangements of α- and β-chitin resulted in distinct reinforcement mechanisms in electrospun PLLA composites. α-Chitin formed a more constrained interfacial environment associated with enhanced stiffness and yield strength, whereas β-chitin promoted a more heterogeneous local molecular environment that preserved greater chain mobility and facilitated post-yield deformation. The DSC results further suggest a limited local nucleation effect associated with β-chitin, despite the lower overall crystallinity of the composite. These distinct molecular environments provide a structural basis for the greater ductility and toughness observed in PLLA/β-chitin. This structure–property relationship offers a practical strategy for tuning the mechanical performance of PLLA-based nanofiber composites. The utilization of α-chitin is recommended in scenarios where elevated yield strength is imperative, while β-chitin is advised for applications requiring a synergy of toughness and ductility. This recommendation holds particular relevance in the context of biomedical scaffolds, flexible packaging, and other load-bearing biodegradable materials.

## Figures and Tables

**Table 1:** Thermal and crystallization parameters obtained from DSC analysis.

Sample	T_g_ (°C)	T_cc_ (°C)	T_m_ (°C)	X_c_ (%)
PLLA	64	79	179	49
PLLA/α-chitin	64	77	177	23
PLLA/β-chitin	64	88	175 (181) ^a^	14

^a^ Melting temperature of shoulder; T_g_: glass transition temperature; T_cc_: cold-crystallization temperature; T_m_: melting temperature; X_c_: degree of crystallinity.

**Table 2:** Characteristic FT-IR band assignments and wavenumbers.

Assignment	α-Chitin (cm^−1^)	β-Chitin (cm^−1^)	PLLA (cm^−1^)	PLLA/α-Chitin (cm^−1^)	PLLA/β-Chitin (cm^−1^)
C–H stretching (asym.)	-	-	2998	2998	2998
C–H stretching (sym.)	2874	2875	2945	2945	2945
C–H bending	1428	1428	1454	1454	1454
C–H bending	-	-	1362	1362	1362
C=O stretching	-	-	1749	1755	1755
C–O stretching	1026	1026	1180	1185	1185
C–OH bending	-	-	1081	1081	1065
C–COO stretching	-	-	867	867	-
O–H stretching	3435	3435	-	3438	3438
N–H stretching	3268	3270	-	3267	3267
Amide I	1624	1625	-	1626	1626
Amide II	1550	1552	-	1552	1552

## Data Availability

Data are contained within the article. The original contributions presented in this study are included in the article. Further inquiries can be directed to the corresponding author.

## References

[1] Yang F., Murugan R., Ramakrishna S., Wang X., Ma Y.-X., Wang S. (2004). Fabrication of nano-structured porous PLLA scaffold intended for nerve tissue engineering. Biomaterials.

[2] Babichuk I.S., Lin C., Qiu Y., Zhu H., Ye T.T., Gao Z., Yang J. (2022). Raman mapping of piezoelectric poly (l-lactic acid) films for force sensors. RSC Adv..

[3] Liao J., Zhou Y., Hou B., Zhang J., Huang H. (2023). Nano-Chitin: Preparation strategies and food biopolymer film reinforcement and applications. Carbohydr. Polym..

[4] Nagarajan V., Mohanty A.K., Misra M. (2016). Perspective on polylactic acid (PLA) based sustainable materials for durable applications: Focus on toughness and heat resistance. ACS Sustain. Chem. Eng..

[5] Prabhakaran M.P., Venugopal J., Ramakrishna S. (2009). Electrospun nanostructured scaffolds for bone tissue engineering. Acta Biomater..

[6] Lee Y.H., Lee J.H., An I.-G., Kim C., Lee D.S., Lee Y.K., Nam J.-D. (2005). Electrospun dual-porosity structure and biodegradation morphology of Montmorillonite reinforced PLLA nanocomposite scaffolds. Biomaterials.

[7] Capuana E., Lopresti F., Ceraulo M., La Carrubba V. (2022). Poly-l-lactic acid (PLLA)-based biomaterials for regenerative medicine: A review on processing and applications. Polymers.

[8] Renouf-Glauser A.C., Rose J., Farrar D.F., Cameron R.E. (2005). The effect of crystallinity on the deformation mechanism and bulk mechanical properties of PLLA. Biomaterials.

[9] Gay S., Arostegui S., Lemaitre J. (2009). Preparation and characterization of dense nanohydroxyapatite/PLLA composites. Mater. Sci. Eng. C.

[10] Liu H., Zhang J. (2011). Research progress in toughening modification of poly (lactic acid). J. Polym. Sci. B Polym. Phys..

[11] Park Y., Lee J. (2023). Comparison of recently developed toughening strategies for polylactic acid blends. J. Ind. Eng. Chem..

[12] Li T., Zhang J., Schneiderman D.K., Francis L.F., Bates F.S. (2016). Toughening glassy poly(lactide) with block copolymer micelles. ACS Macro Lett..

[13] Alias N.F., Ismail H. (2019). An overview of toughening polylactic acid by an elastomer. Polym.-Plast. Technol. Mater..

[14] Niu G., Tao Y., Zhang R., Feng X., Deng H., Ren M., Sun J., Peijs T. (2024). Toughening of self-reinforced PLA films using PLA nanofiber mats and oriented PLA tapes as interlayers. Compos. Part A Appl. Sci. Manuf..

[15] Cai N., Dai Q., Wang Z., Luo X., Xue Y., Yu F. (2015). Toughening of electrospun poly(l-lactic acid)nanofiber scaffolds with unidirectionally aligned halloysite nanotubes. J. Mater. Sci..

[16] Liu Y., Wei H., Wang Z., Li Q., Tian N. (2018). Simultaneous enhancement of strength and toughness of PLA induced by miscibility variation with PVA. Polymers.

[17] Zhang Q., Mochalin V.N., Neitzel I., Hazeli K., Niu J., Kontsos A., Zhou J.G., Lelkes P.I., Gogotsi Y. (2012). Mechanical properties and biomineralization of multifunctional nanodiamond-PLLA composites for bone tissue engineering. Biomaterials.

[18] Li C., Shi B., Jiang J., Lin H., Xu Y., Lin S., Zhao L., Meng X., Xi Z. (2025). Revisiting the effect of constructing stereocomplex crystallites through a compatibilizer in super tough PLLA blends. Macromolecules.

[19] Rinaudo M. (2006). Chitin and chitosan: Properties and applications. Prog. Polym. Sci..

[20] Jang M.K., Kong B.G., Jeong Y.I., Lee C.H., Nah J.W. (2004). Physicochemical characterization of α-chitin, β-chitin, and γ-chitin separated from natural resources. J. Polym. Sci. Part A Polym. Chem..

[21] Hajji S., Younes I., Ghorbel-Bellaaj O., Hajji R., Rinaudo M., Nasri M., Jellouli K. (2014). Structural differences between chitin and chitosan extracted from three different marine sources. Int. J. Biol. Macromol..

[22] Li X., Feng Q., Cui F. (2006). In vitro degradation of porous nano-hydroxyapatite/collagen/PLLA scaffold reinforced by chitin fibres. Mater. Sci. Eng. C.

[23] Olaiya N., Maraveas C., Salem M.A., Raja S., Rashedi A., Alzahrani A.Y., El-Bahy Z.M., Olaiya F.G. (2022). Viscoelastic and properties of amphiphilic chitin in plasticised polylactic acid/starch biocomposite. Polymers.

[24] Choy S., Moon H., Park Y., Jung Y.M., Koo J.M., Oh D.X., Hwang D.S. (2020). Mechanical properties and thermal stability of intermolecular-fitted poly(vinyl alcohol)/α-chitin nanofibrous mat. Carbohydr. Polym..

[25] Moon H., Choy S., Park Y., Jung Y.M., Koo J.M., Hwang D.S. (2019). Different molecular interaction between collagen and α- or β-chitin in mechanically improved electrospun composite. Mar. Drugs.

[26] Kalb B., Pennings A.J. (1980). General crystallization behaviour of poly(L-lactic acid). Polymer.

[27] Hassan M.M., Koyama K. (2020). Thermomechanical and viscoelastic properties of green composites of PLA using chitin micro-particles as fillers. J. Polym. Res..

[28] Jędrzejczak E., Frąckowiak P., Sibillano T., Brendler E., Giannini C., Jesionowski T., Wysokowski M. (2024). Isolation and Structure Analysis of Chitin Obtained from Different Developmental Stages of the Mulberry Silkworm (*Bombyx mori*). Molecules.

[29] Tsurkan M.V., Voronkina A., Khrunyk Y., Wysokowski M., Petrenko I., Ehrlich H. (2021). Progress in chitin analytics. Carbohydr. Polym..

[30] Hou J., Aydemir B.E., Dumanli A.G. (2021). Understanding the structural diversity of chitins as a versatile biomaterial. Philos. Trans. A Math. Phys. Eng. Sci..

[31] Richard-Lacroix M., Pellerin C. (2013). Molecular orientation in electrospun fibers: From mats to single fibers. Macromolecules.

[32] Noishiki Y., Takami H., Nishiyama Y., Wada M., Okada S., Kuga S. (2003). Alkali-induced conversion of β-chitin to α-chitin. Biomacromolecules.

[33] Greenfeld I., Sui X., Wagner H.D. (2016). Stiffness, strength, and toughness of electrospun nanofibers: Effect of flow-induced molecular orientation. Macromolecules.

[34] Chieng B.W., Ibrahim N.A., Wan Yunus W.M.Z., Hussein M.Z. (2013). Poly(lactic acid)/Poly(ethylene glycol) polymer nanocomposites: Effects of graphene nanoplatelets. Polymers.

[35] Kia M.V., Ehsani M., Hosseini S.E., Asadi G.H. (2024). Fabrication and characterization of transparent nanocomposite films based on poly (lactic acid)/polyethylene glycol reinforced with nano glass flake. Int. J. Biol. Macromol..

[36] Gupta A., Pal A.K., Woo E.M., Katiyar V. (2018). Effects of amphiphilic chitosan on stereocomplexation and properties of poly(lactic acid) nano-biocomposite. Sci. Rep..

[37] Hsieh Y.-T., Nozaki S., Kido M., Kamitani K., Kojio K., Takahara A. (2020). Crystal polymorphism of polylactide and its composites by X-ray diffraction study. Polym. J..

